# Venovenous Extracorporeal Membranous Oxygenation for Tracheoparenchymal Trauma: A Case Report

**DOI:** 10.7759/cureus.117369

**Published:** 2026-10-02

**Authors:** Sydney R Davis, Lauren A Bitterman, Alexander N Sumarli

**Affiliations:** 1 Anesthesiology, Loma Linda University, Loma Linda, USA

**Keywords:** airway surgeries, penetrating thoracic trauma, thoracic anesthesiology, tracheal injury, venovenous extracorporeal membrane oxygenation

## Abstract

Penetrating tracheal injuries present unique challenges for airway management, ventilation, and surgical repair due to the risk of rapid airway decline. We present a case of a 34-year-old male who sustained an arrow injury to the neck. Given the risk of airway compromise with induction or movement, venovenous extracorporeal membrane oxygenation (VV ECMO) was initiated prior to anesthesia. Awake bronchoscopy confirmed tracheal injury with an embedded arrow. The patient underwent successful tracheal repair and robotic arrow extraction under ECMO support. He was weaned off ECMO intraoperatively and discharged without complications. This case highlights the utility of early VV ECMO as a bridge to definitive airway and surgical management in complex penetrating airway trauma.

## Introduction

Penetrating tracheal injuries are rare, but pose significant challenges for airway management, ventilation, and surgical repair. Tracheal and tracheobronchial injuries may result from blunt trauma, penetrating wounds, or iatrogenic causes, including endotracheal intubation or endobronchial interventions. The precise incidence of these injuries is unknown. One study reported 1,033 tracheal injuries over a five-year period, including 429 non-iatrogenic and 604 iatrogenic injuries, while another review identified 265 cases of blunt intrathoracic tracheobronchial injury [1,2]. Even small tracheal injuries can lead to rapid airway decompensation [3]. Intubation and ventilation can risk exacerbating a tracheal injury, creating false passages, or result in respiratory failure [4,5]. Extracorporeal membrane oxygenation (ECMO) is an important option to consider in managing complex airway trauma. ECMO provides a means to maintain oxygenation and ventilation without manipulating a tracheal injury prior to surgical repair. Although ECMO is well described in severe respiratory failure, reports describing its use for penetrating tracheal trauma remain limited. This case presents the successful use of early venovenous (VV) ECMO to facilitate safe bronchoscopy, surgical exposure, and robotic extraction of a retained arrow traversing the trachea and thoracic cavity. We aim to highlight the role of ECMO as a bridge to definitive airway control and repair in high-risk airway injuries to minimize perioperative risk and improve patient outcomes.

## Case presentation

A 34-year-old, 108 kg, and 175 cm tall male with no known medical history was brought to the emergency department after sustaining an arrow injury to his midline neck while loading his crossbow at home. He reported removing the exposed portion of the arrow himself prior to arrival, however the arrowhead and a portion of the arrow remained within the neck and thorax. On physical exam, he had a small 1 cm midline anterior neck wound with no active bleeding but noticeable crepitus. He was breathing spontaneously on room air without signs of respiratory insufficiency. His neurologic examination was normal; he was conversing normally and fully oriented. Computed tomography (CT) of his chest revealed a 7 cm arrow with trajectory through the anterior midline chest into the superior mediastinum terminating within the right superior lower lobe (Figure 1, Figure 2). Laboratory values including complete blood count and comprehensive metabolic panel were within normal limits (Table 1). High-sensitivity troponin count was <6 ng/L, which was within normal limits. Additionally, other imaging including chest X-ray showed an associated small right apical pneumothorax, small volume pneumomediastinum, subcutaneous emphysema, and a small right upper lobe contusion or laceration (Figure 3). There was no evidence of active arterial extravasation or mediastinal shift.

**Figure 1:**
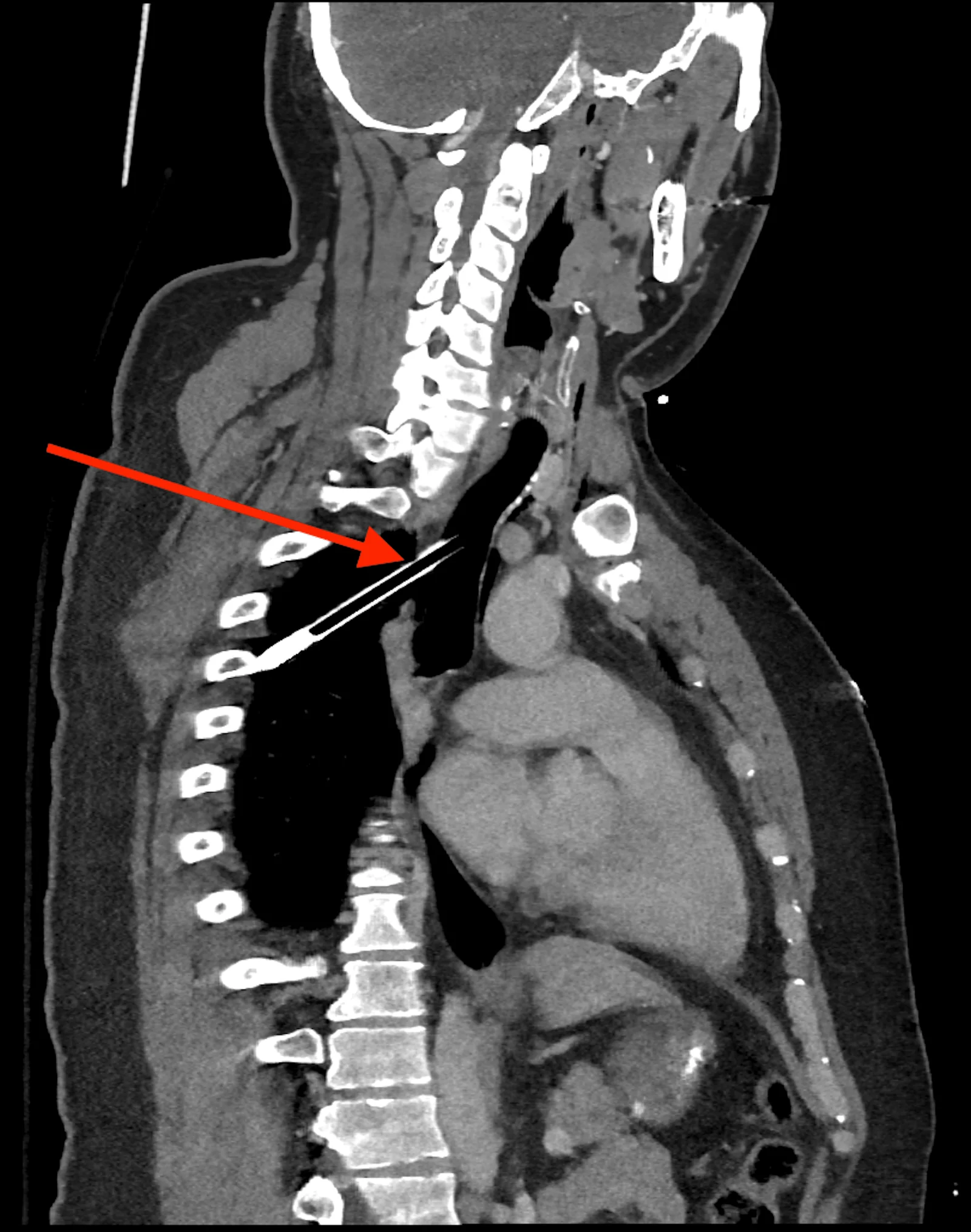
CT multiplanar reconstruction along the sagittal plane of the missile, showing extension through the trachea (red arrow).

**Figure 2:**
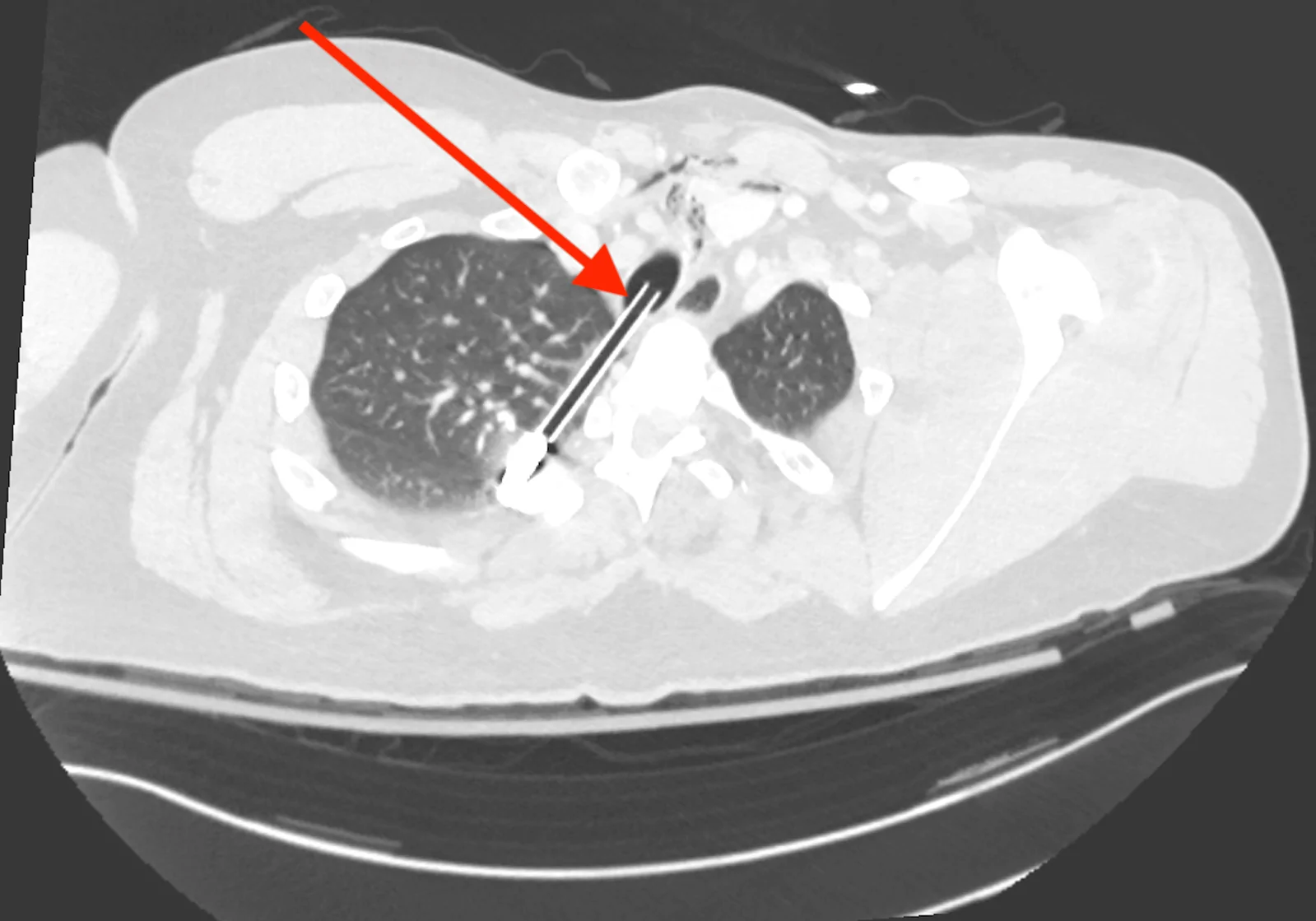
CT multiplanar reconstruction along the axial plane of the missile, showing extension through the trachea (red arrow).

**Table 1:** Laboratory tests on admission. WBC: white blood cell count, Hgb: hemoglobin, Hct: hematocrit, PT: prothrombin time, INR: international normalized ratio, PTT: partial thromboplastin time, BUN: blood urea nitrogen, AST: aspartate aminotransferase, ALT: alanine aminotransferase

Parameter	Result	Reference Range
WBC (bil/L)	13.46	4.80-11.80
Hgb (g/dL)	15.1	11.0-16.0
Hct (%)	43.6	33.5-47.0
Platelets (bil/L)	232	140-340
PT (sec)	10.8	9.4-12.5
INR	0.9	0.8-1.1
PTT (sec)	30.7	25.1-36.5
Fibrinogen (mg/dL)	267	200-393
Sodium (mMol/L)	140	134-147
Potassium (mMol/L)	3.8	3.5-5.0
Chloride (mMol/L)	106	98-109
CO_2_ (mMol/L)	22	23-32
Anion Gap	13	7-16
BUN (mg/dL)	8	7-20
Creatinine (mg/dL)	0.9	0.7-1.3
AST (U/L)	22	0-35
ALT (U/L)	21	7-45
Alkaline phosphatase (U/L)	89	37-132
Total Bilirubin (mg/dL)	0.3	0-1.0
Lactate, Vein (mMol/L)	2.6	0.5-2.0

**Figure 3:**
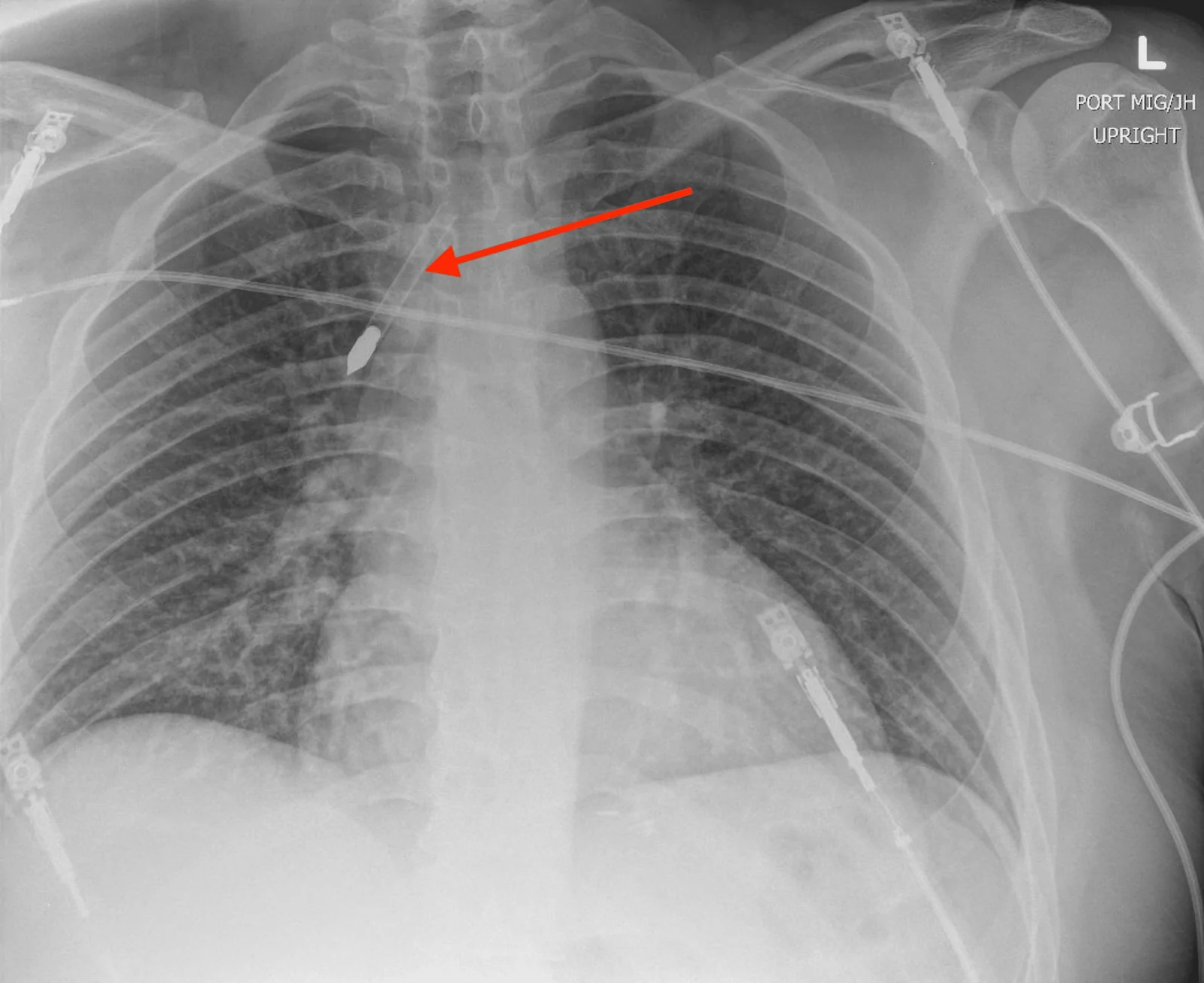
Plain film chest X-ray showing missile embedded within the right upper lung parenchyma with extension into the trachea (red arrow).

At the onset of the case, discussion occurred between anesthesiology, cardiothoracic surgery, the ECMO team, and trauma surgery to formulate a surgical and airway plan. Given concern for the arrow remaining lodged in the trachea and the arrowhead in the right pleural cavity with the possibility of surrounding injury in the case of dislodgement with intubation or patient positioning, VV ECMO cannulation was performed prior to induction. A 21 Fr cannula was inserted into the right common femoral vein, with its tip positioned at the level of the right atrium and confirmed by X-ray. Another 25 Fr venous cannula was placed into the left common femoral vein, with the cannula tip positioned approximately 10 cm distal from the first cannula. A pre-induction arterial line and a right internal jugular vein central line were also placed. The patient was then initiated on VV ECMO. Awake bronchoscopy under mild sedation while maintaining spontaneous ventilation was subsequently performed showing two injuries: one superficial anterior tracheal injury approximately 4 cm distal to the vocal cords and one right posterolateral tracheal injury with part of the arrow embedded into the trachea, approximately 5 cm from the carina. A 6.0 microlaryngeal tube (MLT) was placed proximal to the level of the injuries with a fiberoptic scope, however the ventilator was subsequently turned off and paralysis was maintained after a satisfactory level of anesthesia was reached to minimize respirophasic movements. Maintenance of anesthesia was achieved with total intravenous anesthesia with propofol and remifentanil infusions. Initially, the patient desaturated to SpO2 of 87% and ECMO flow rate was increased to 5 liters per minute. His ECMO at this point did not have adequate venous drainage, causing the chatter phenomenon, in which 200 mL of albumin was then administered as volume support. His oxygen saturation improved to SpO2 >90% throughout the remainder of the case without ventilator support.

To minimize positioning changes of the patient, the trauma surgery team first performed an anterior repair of the neck and the trachea. An esophagogastroduodenoscopy (EGD) was then performed with no evidence of esophageal injury. The patient was then moved into the left lateral decubitus position with brief desaturation to 80%. Additionally, the chatter phenomenon on ECMO was again observed, which improved with an additional 250 mL of albumin administered for volume support. A robotic-assisted thoracoscopic extraction of the arrow from the right upper lobe was performed. The arrowhead was found penetrating the trachea behind the superior vena cava, above the azygos vein, anterior to the esophagus. The missile was extracted intact. Repeat bronchoscopy after tracheal repair showed good approximation of the tracheal injury with no air leak seen on the ventilator. The patient was then subsequently weaned from VV ECMO with the 6.0 MLT in place. He was successfully extubated and transferred to recovery with oxygen via a face mask with a chest tube in place. He was admitted to the surgical intensive care unit (ICU) for airway monitoring, then subsequently had his chest tube removed on post-operative day (POD) 4. The patient was discharged from the hospital shortly thereafter.

## Discussion

Tracheal injuries can be a significant challenge for airway management and ventilation. The primary concerns in such cases of tracheal injury typically include persistent air leaks, airway instability, pneumomediastinum or subcutaneous emphysema, and progression to respiratory failure [3]. Given the potential for rapid deterioration, particularly during induction of anesthesia and intubation, a multidisciplinary approach and consideration of alternative oxygenation strategies is necessary. The use of ECMO should be considered in patients with severe airway obstruction due to anterior neck or tracheal disease or injury [6]. This discussion reviews airway management options for tracheal trauma, the utility of ECMO as a bridge to definitive care, and the rationale for early ECMO initiation as presented in our case.

In cases involving airway injury, there are several approaches to consider. Blind endotracheal intubation can lead to worsening of the tracheal injury, with widening of the laceration or even formation of a false passage [4]. Paralysis for intubation can lead to tracheal collapse, making awake intubation ideal for certain patients. Awake placement of an endotracheal tube using a flexible bronchoscope and inflation of the balloon distal to the site of injury can help limit apnea and loss of smooth muscle tone from paralysis [5]. When the patient is rapidly desaturating, hemodynamically unstable, or is not tolerating an awake intubation, rigid bronchoscopy while the patient receives inhalational induction may be the method of choice [5]. Tracheostomy is not routinely performed in cases of tracheal trauma, but can be considered in patients with craniomaxillofacial injuries or when previous endotracheal intubation attempts were unsuccessful [7]. In cases where effective ventilation cannot be guaranteed or in challenging cases, ECMO support may be considered as a useful tool.

ECMO is a form of advanced cardiopulmonary life support that can be configured in different ways depending on the clinical indication. In VV ECMO, blood is drained from the venous system and returned in the venous system, primarily supporting respiratory function. In veno-arterial (VA) ECMO, blood is drained from the venous system and returned to the arterial system, providing both respiratory and hemodynamic support. ECMO support in tracheo-bronchial surgeries can be a very useful tool to allow for efficient gas exchange without impairment of the surgical field with endotracheal cuffed tubes [8,9]. Currently, an absolute contraindication for initiating ECMO is anticipated nonrecovery without a plan for viable decannulation [10]. ECMO usage requires systemic heparinization which can carry a significant morbidity in certain patients. In patients with traumatic thoracic injury, the risk of heparinization in a patient with potential injuries to the brain, lungs, heart, or thoracic vessels must be considered with careful review of available radiographic imaging and clinical correlation prior to initiation of ECMO [11]. Given ECMO’s ability to provide supportive ventilation and oxygenation in cases where careful airway manipulation is necessary, it serves as a useful bridge to definitive surgical management.

In our patient, the retained foreign body with known penetrating trauma to the trachea and a trajectory extending towards the pleural cavity posed risks to airway instability and potential secondary injury during airway manipulation. Any displacement of the arrow during either intubation or patient positioning into the lateral position carried a risk of exacerbating the underlying injuries or causing new damage to critical structures in the thoracic cavity. Given these concerns, the decision was made to establish ECMO support prior to induction, providing a controlled means of oxygenation and ventilation. Early initiation of VV ECMO can be a critical, life-saving strategy in cases of severe tracheal trauma, especially in cases where endotracheal tube placement may be unsafe or ineffective.

## Conclusions

This case illustrates that early initiation of VV ECMO may be a potential life-saving modality for managing severe penetrating tracheal trauma. By providing controlled oxygenation and ventilation, VV ECMO serves as an effective bridge to definitive surgical repair, allowing clinicians to avoid the risks associated with conventional airway manipulation when the airway is unstable. This multidisciplinary approach minimizes perioperative risk and improves outcomes in complex airway injury scenarios where standard intubation techniques may be unsafe or ineffective.
